# Supplementary figures and images for: Genetic and physical interactions reveal overlapping and distinct contributions to meiotic double-strand break formation in C. elegans
Source: eLife. 2026 Mar 25;13:RP96458. doi: 10.7554/eLife.96458 (PMC13016608; doi:10.7554/eLife.96458)

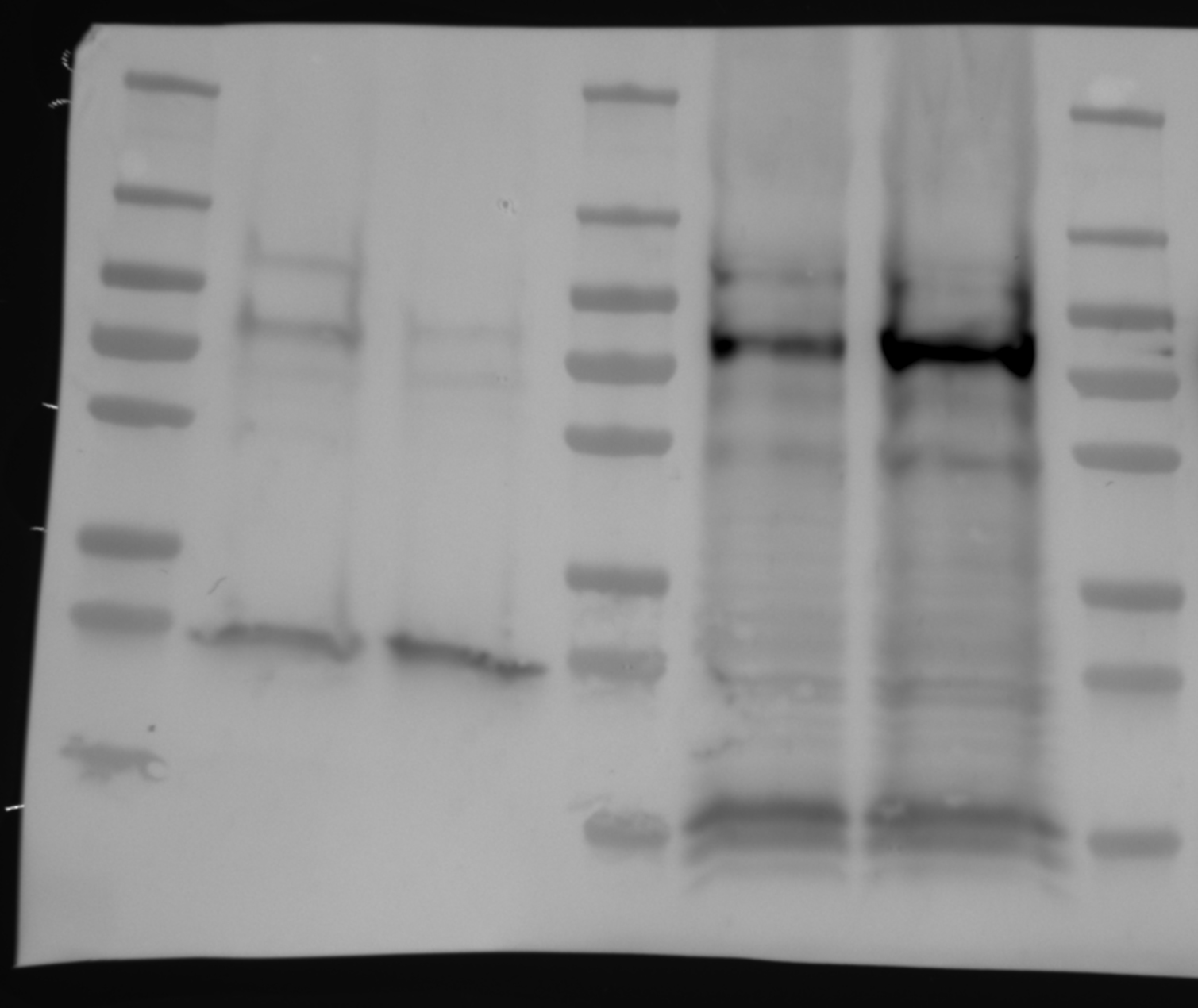

Supplement: Figure 1—source data 1. [file elife-96458-fig1-data1.zip › Figure 1-source data 1/Figure 1-source of data 1.jpg]

him-17::3xHA;  
him-5::GFP::3xFLAG  
him-5::GFP::3x FLAG

him-17::3xHA;  
him-5::GFP::3xFLAG  
him-5::GFP::3x FLAG

XND-1

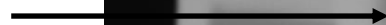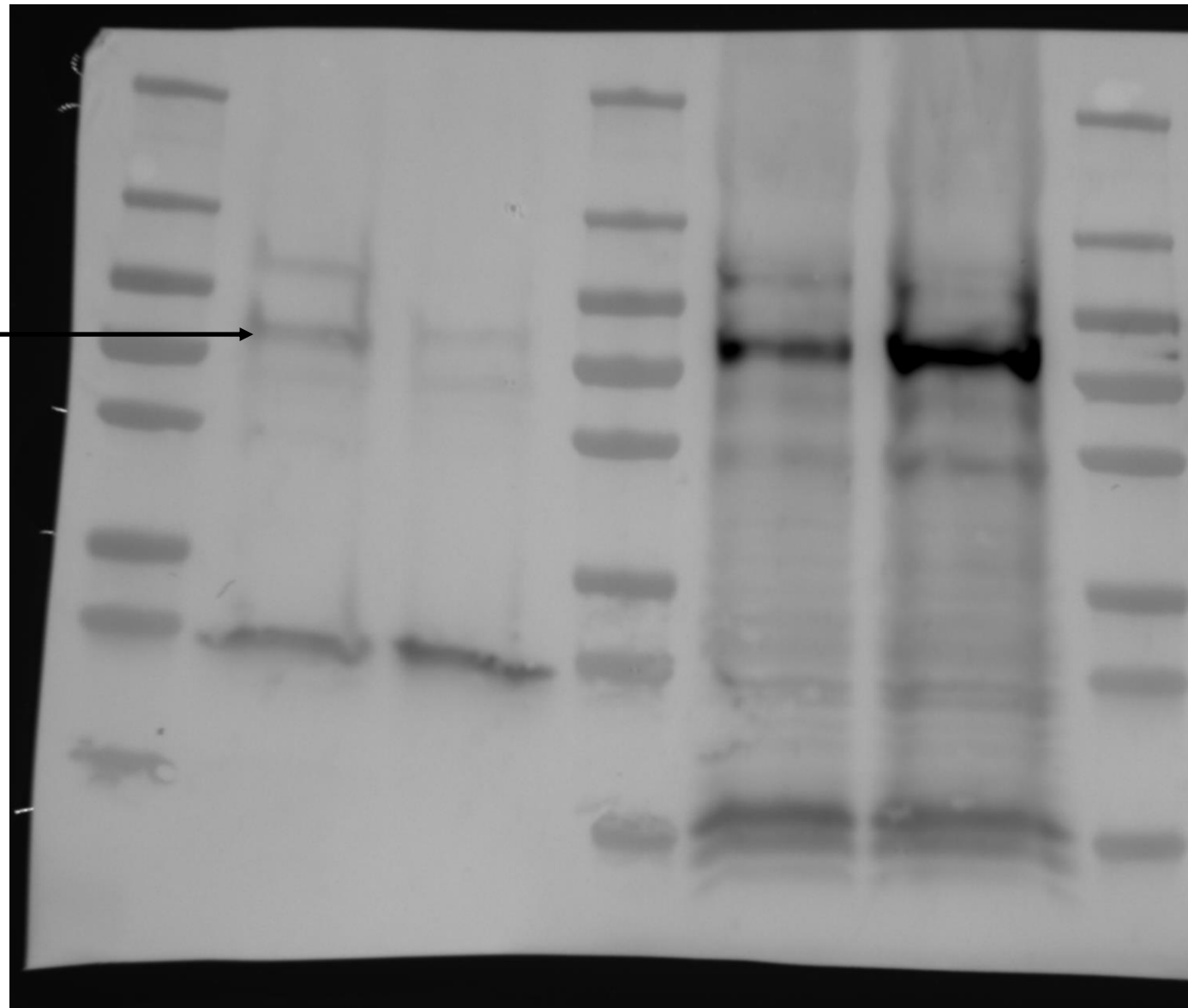

IP

Input (10%)

Supplement: Figure 1—source data 2. [file elife-96458-fig1-data2.zip › Figure 1-source data 2/Figure 1-source data 2.pdf]

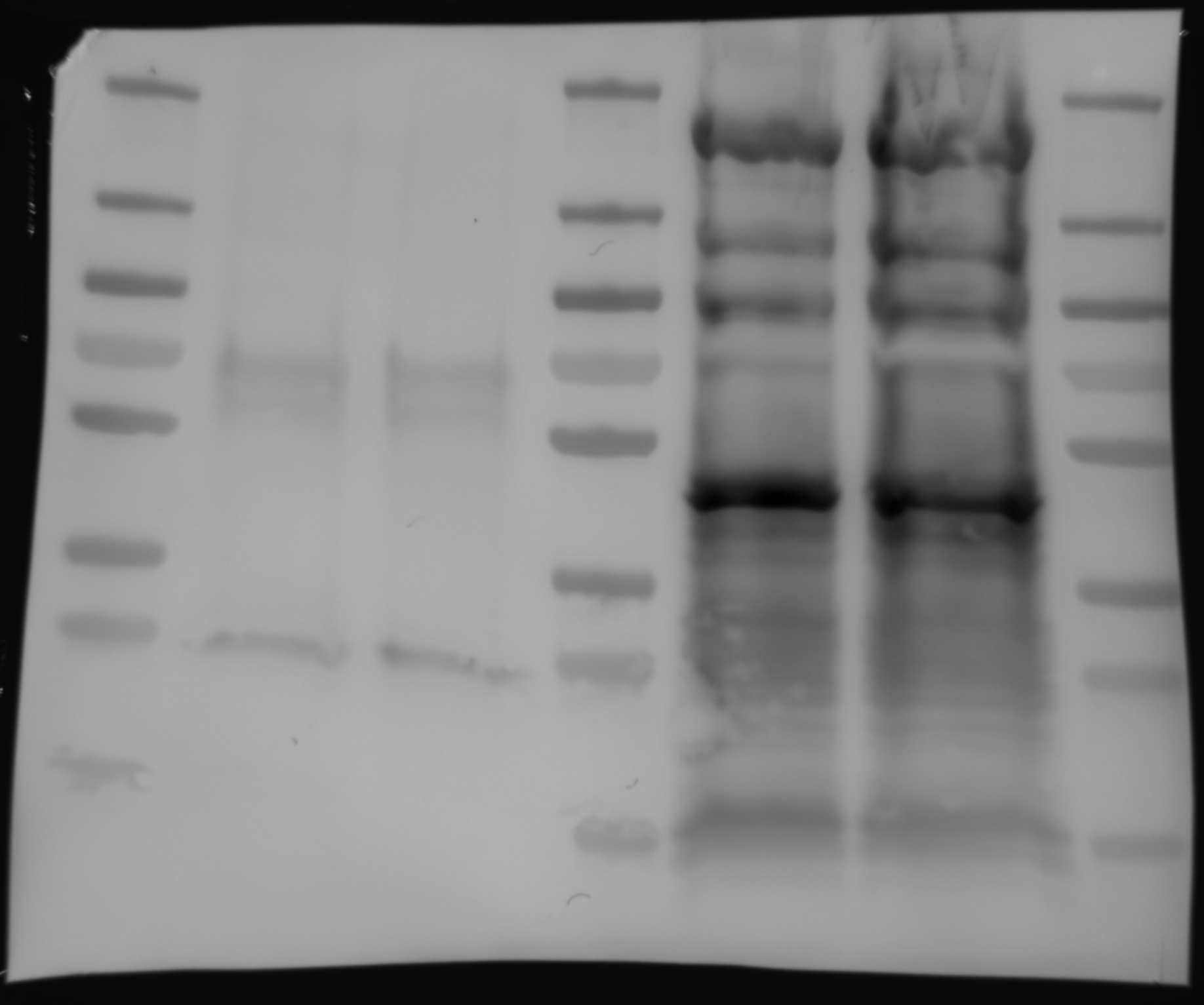

Supplement: Figure 1—source data 3. [file elife-96458-fig1-data3.zip › Figure 1-source data 3/Figure 1-source data 3.jpg]

him-17::3xHA;  
him-5::GFP::3xFLAG

him-5::GFP::3x FLAG

him-17::3xHA;  
him-5::GFP::3xFLAG

him-5::GFP::3x FLAG

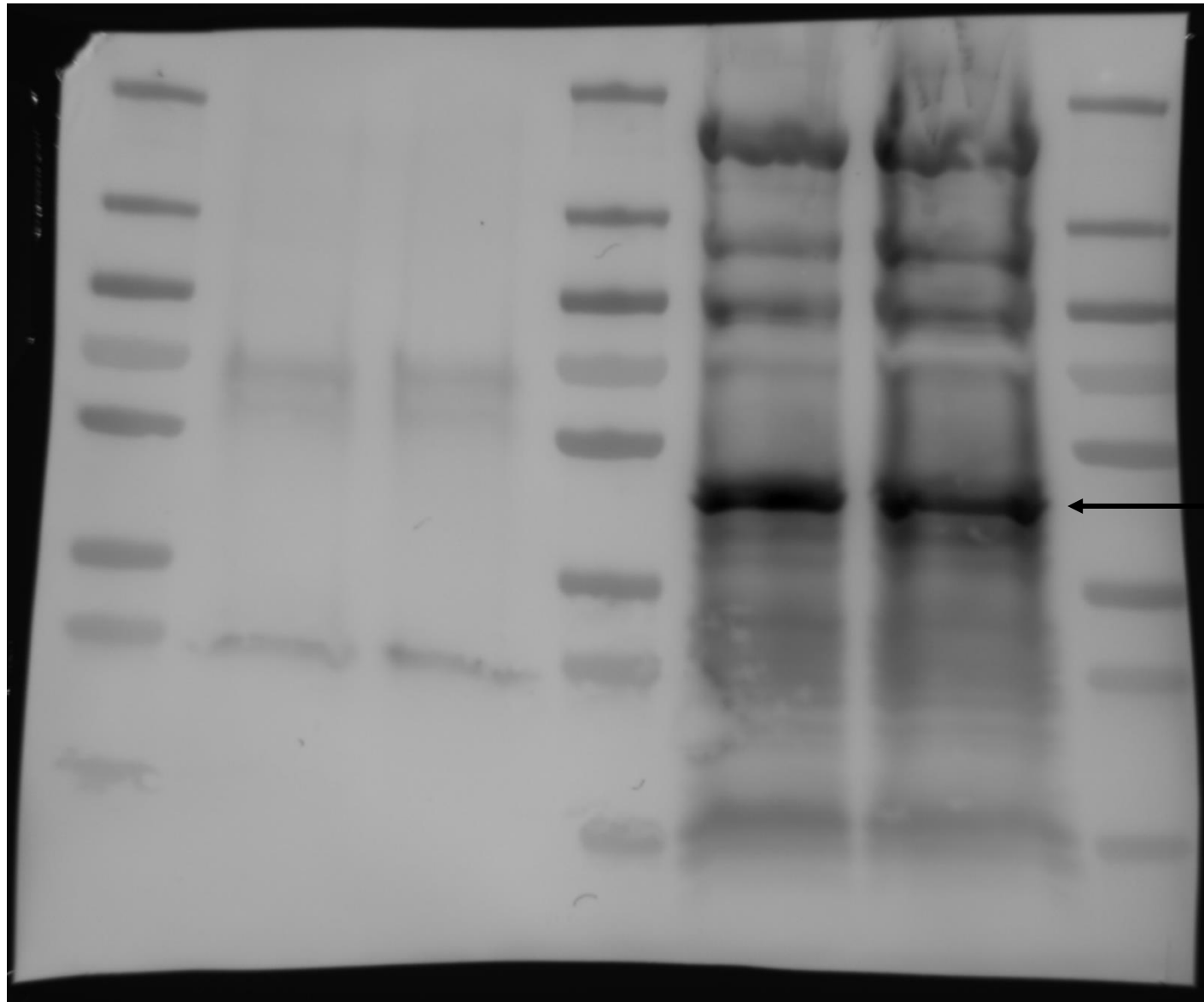

HIM-5

IP

Input (10%)

Supplement: Figure 1—source data 4. [file elife-96458-fig1-data4.zip › Figure 1-source data 4/Figure 1- source data 4.pdf]

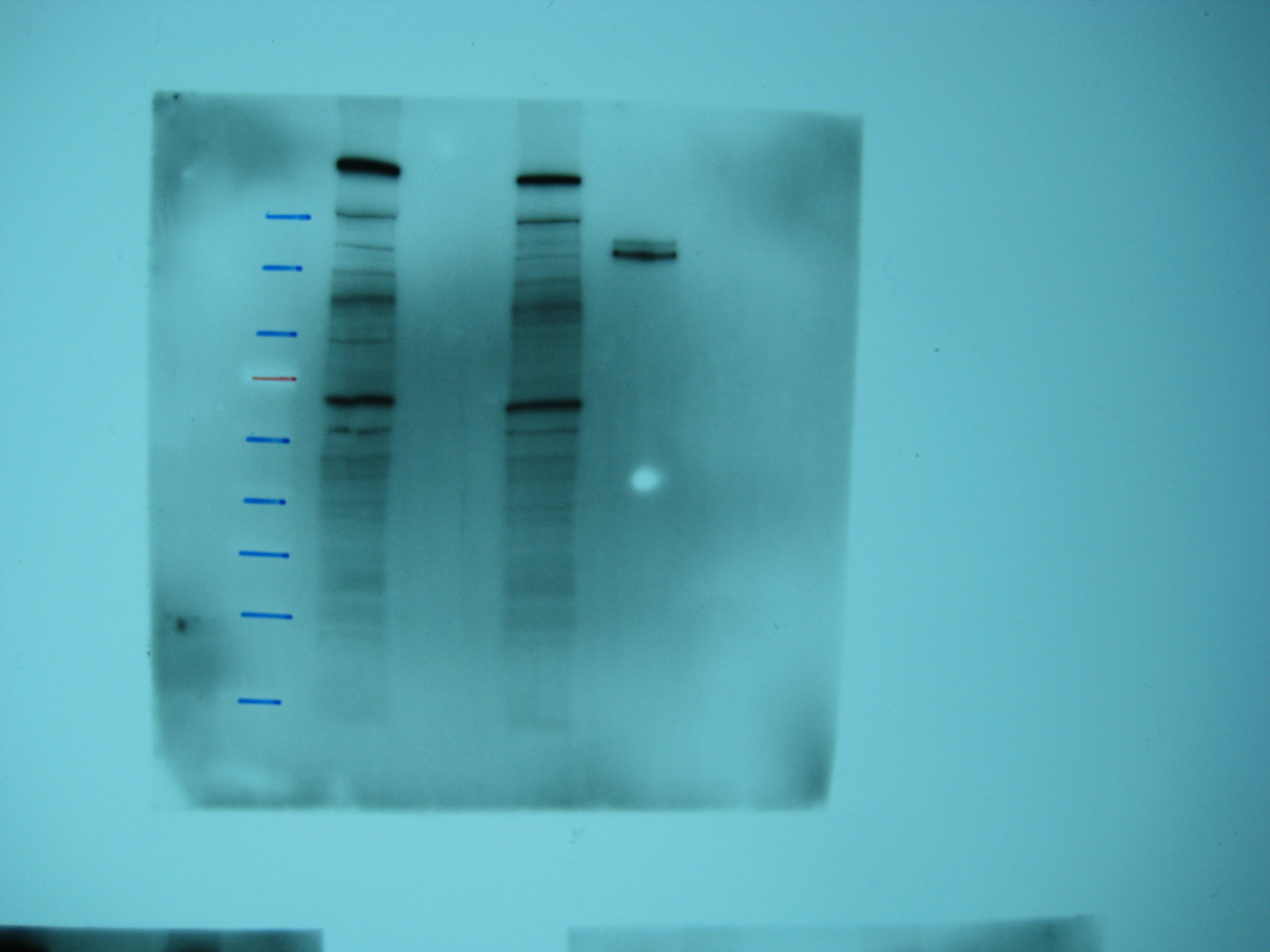

Supplement: Figure 1—figure supplement 1—source data 1. [file elife-96458-fig1-figsupp1-data1.zip › Figure 1-figure supplement 1- source data 1/Figure 1-figure supplement 1- source data 1.jpeg]

WT

HIM-17::GFP

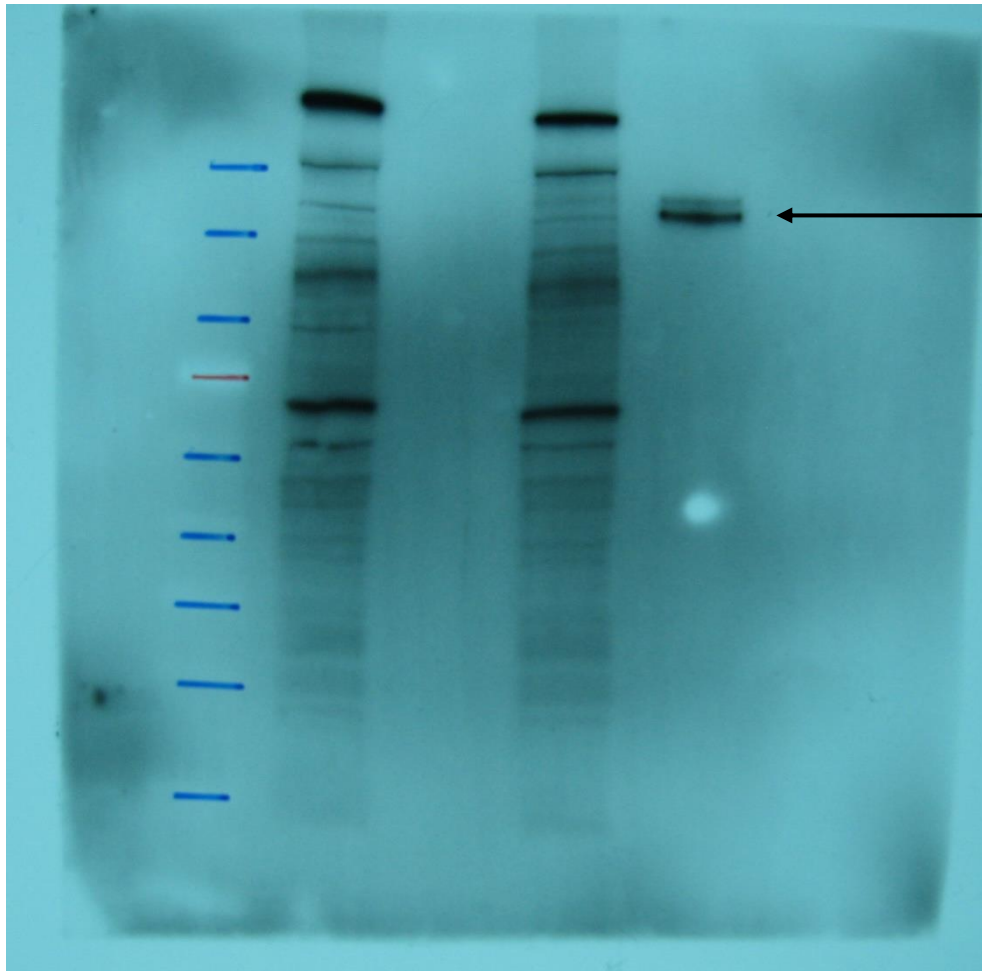

Supplement: Figure 1—figure supplement 1—source data 2. [file elife-96458-fig1-figsupp1-data2.zip › Figure 1-figure supplement 1- source data 2/Figure 1-figure supplement 1-source data 2.pdf]

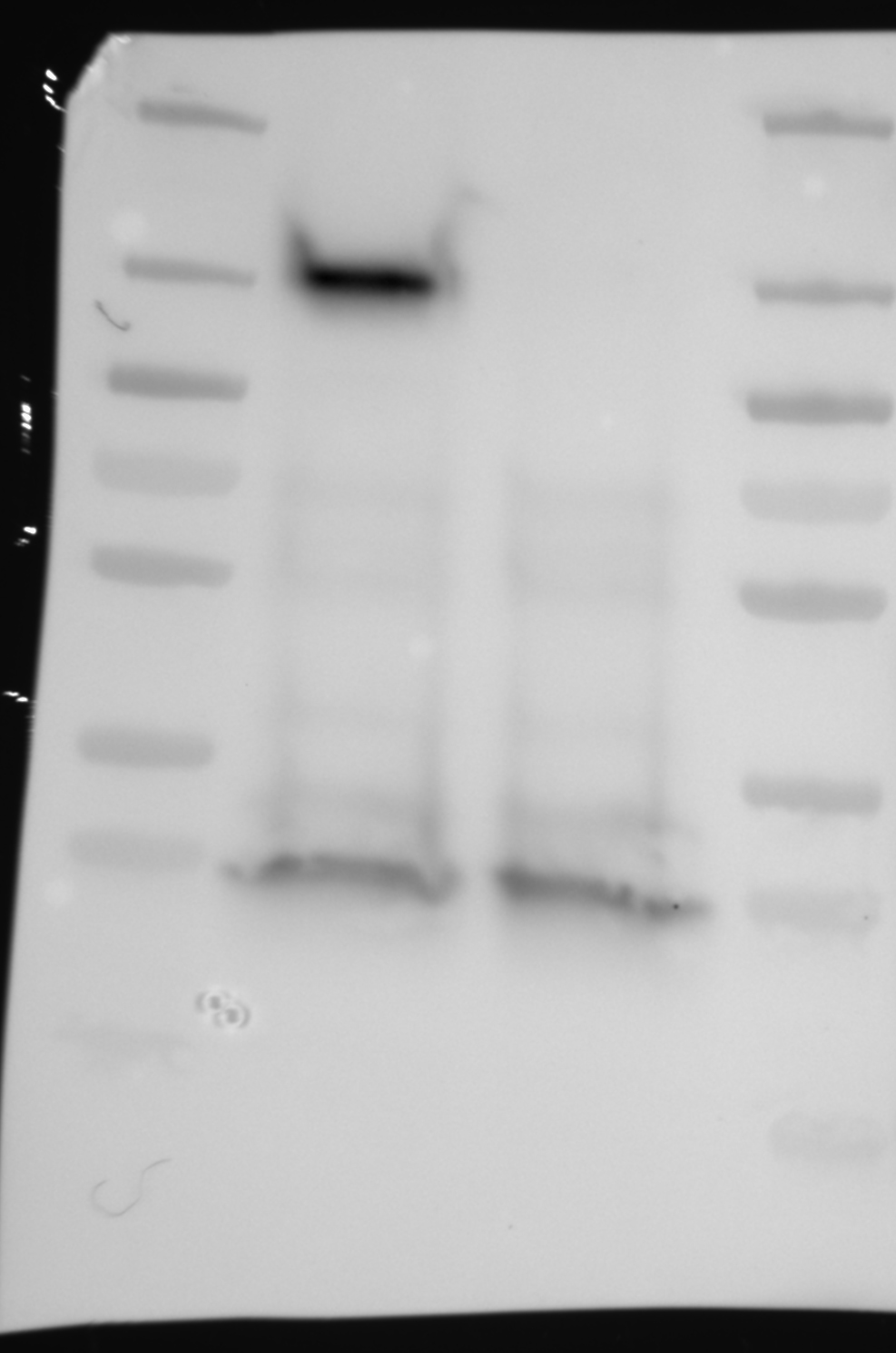

Supplement: Figure 1—figure supplement 1—source data 3. [file elife-96458-fig1-figsupp1-data3.zip › Figure 1-figure supplement 1- source data 3/Figure 1-figure supplement 1-source data 3.jpg]

him-17::3xHA;  
him-5::GFP:3xFLAG  
him-5::GFP:3xFLAG

HIM-17

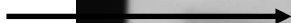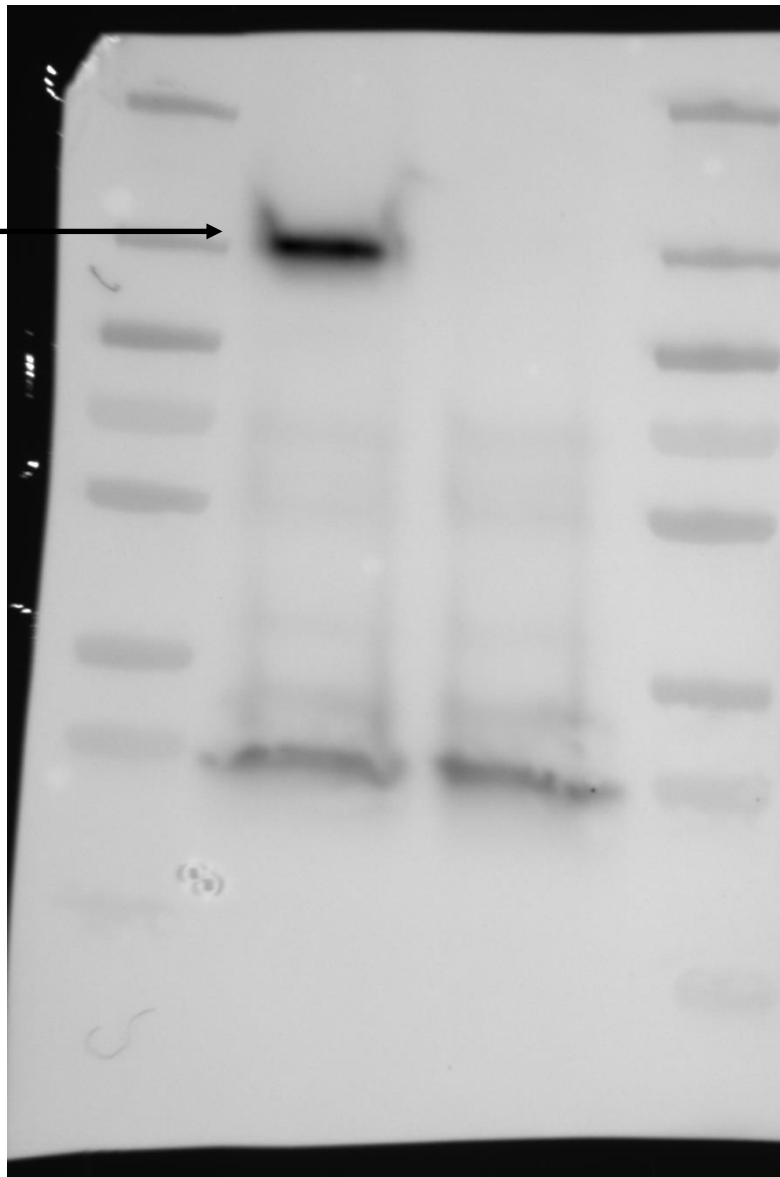

Supplement: Figure 1—figure supplement 1—source data 4. [file elife-96458-fig1-figsupp1-data4.zip › Figure 1-figure supplement 1- source data 4/Figure 1-figure supplement 1- source data 4.pdf]

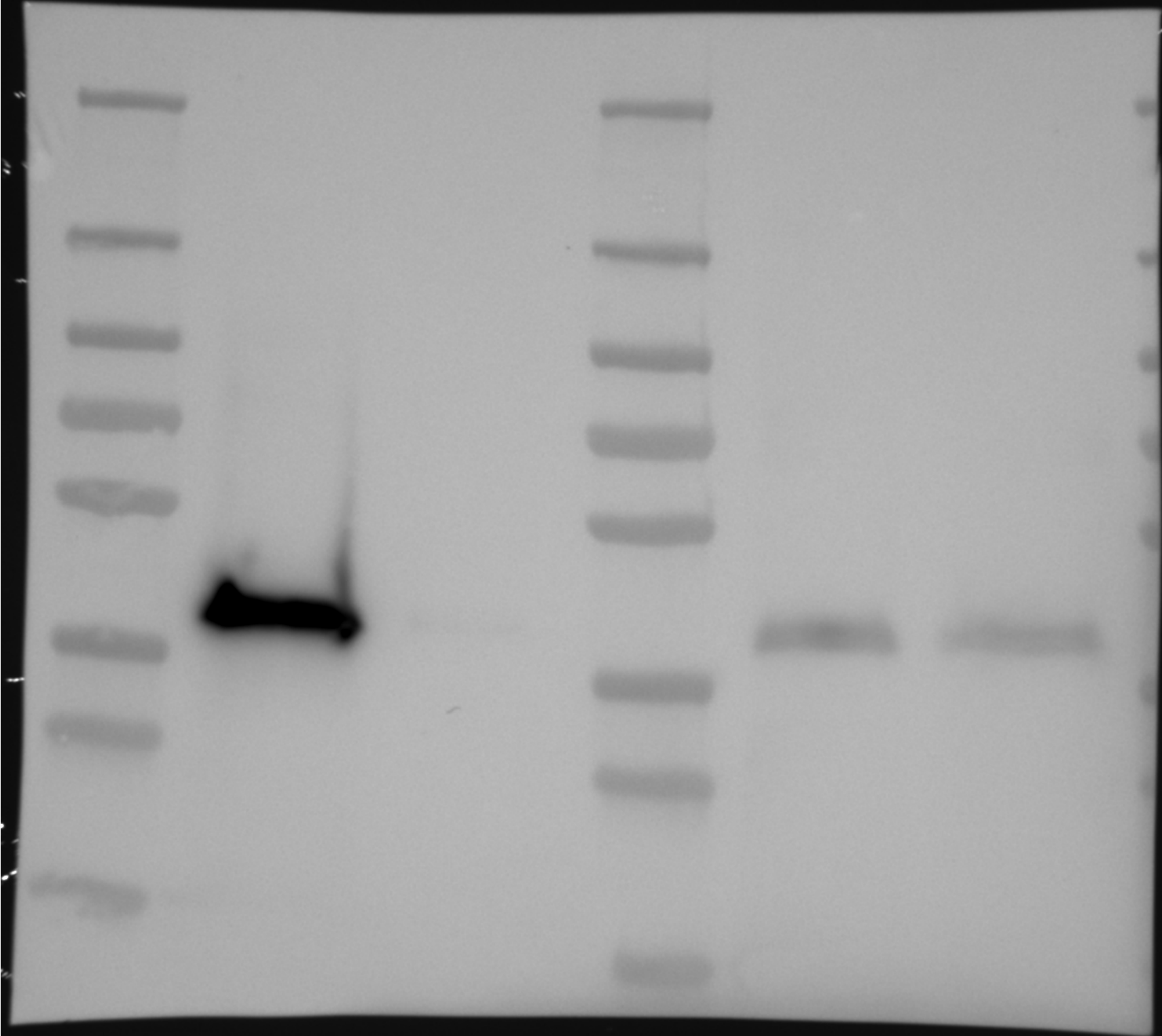

Supplement: Figure 2—source data 1. [file elife-96458-fig2-data1.zip › Figure 2- source data 1/Figure 2- source data 1.pdf]

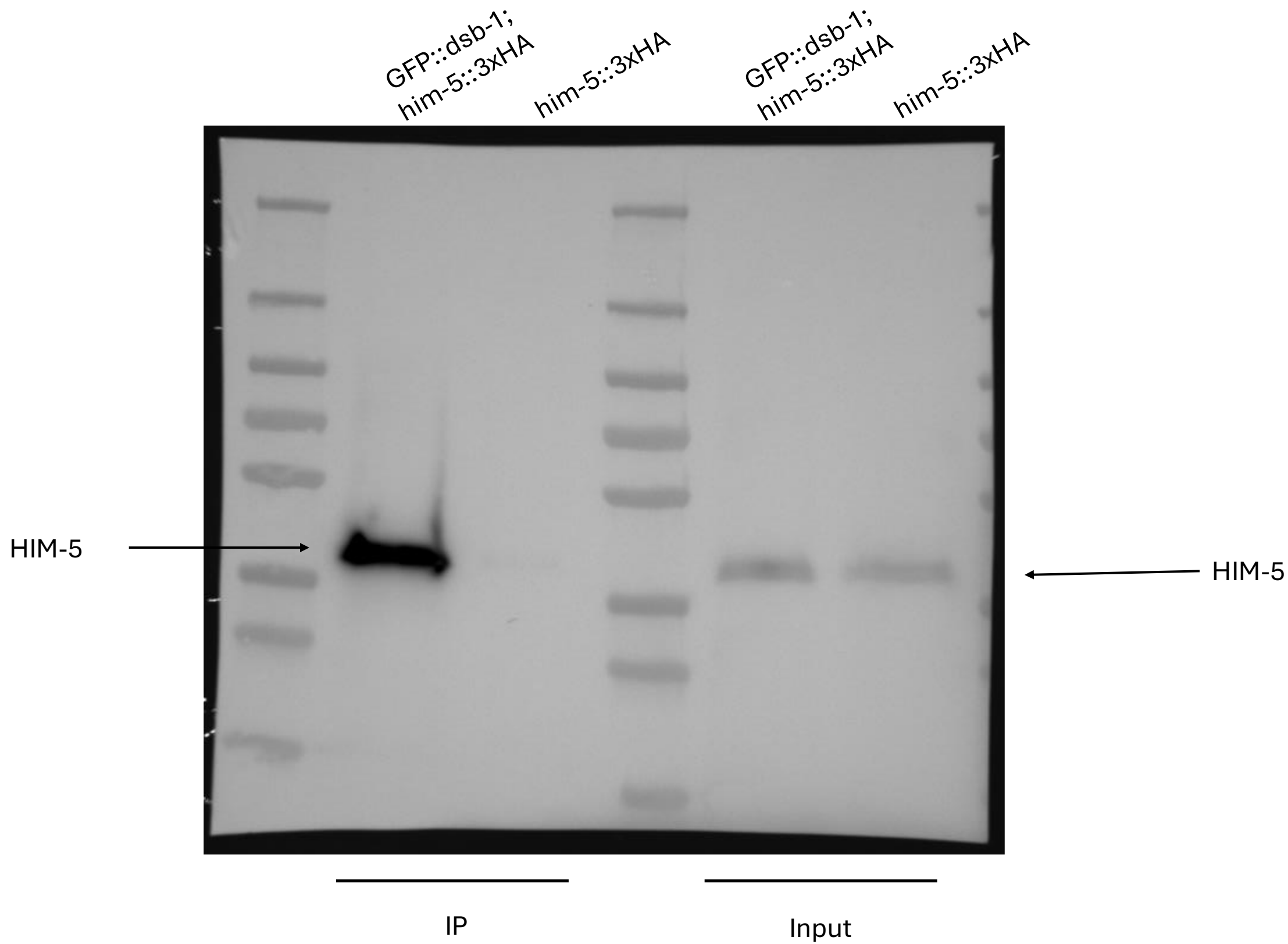

Supplement: Figure 2—source data 2. [file elife-96458-fig2-data2.zip › Figure 2- source data 2/Figure 2- source data 2.pdf]

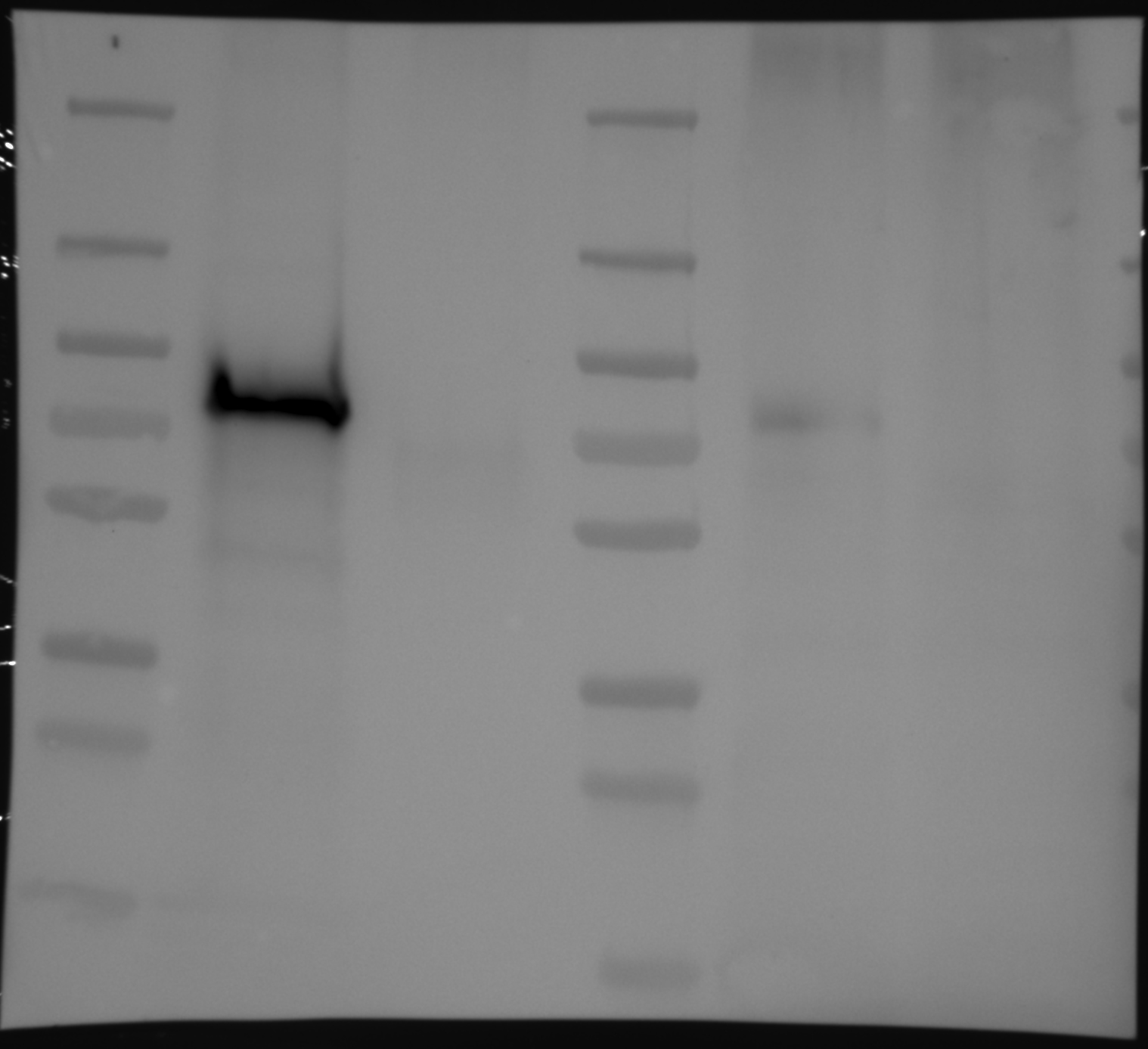

Supplement: Figure 2—source data 3. [file elife-96458-fig2-data3.zip › Figure 2- source data 3/Figure 2-source data 3.jpg]

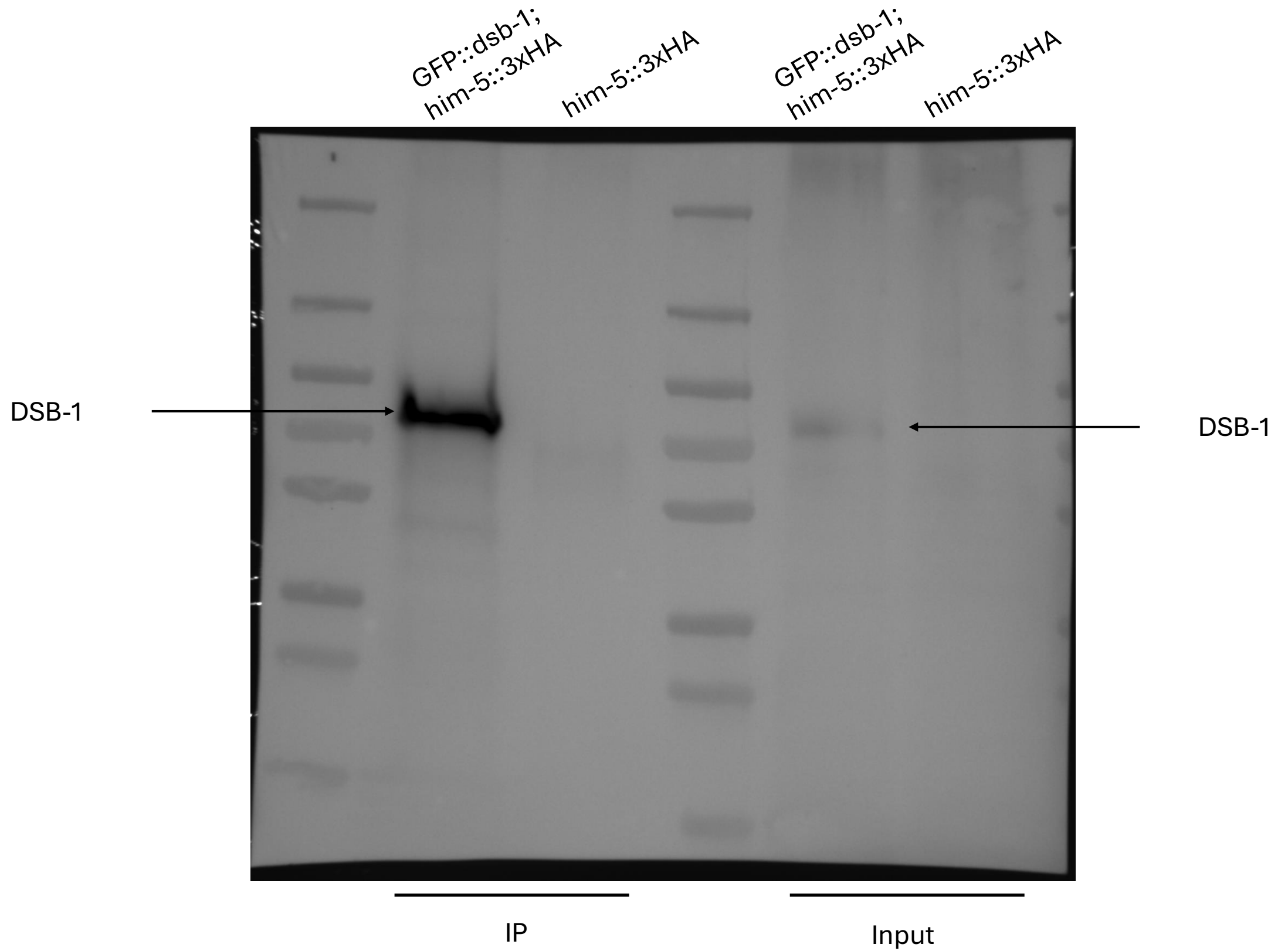

Supplement: Figure 2—source data 4. [file elife-96458-fig2-data4.zip › Figure 2- source data 4/Figure 2- source data 4.pdf]
