# Supplementary material for: Genetic and physical interactions reveal overlapping and distinct contributions to meiotic double-strand break formation in C. elegans
Source: eLife. 2026 Mar 25;13:RP96458. doi: 10.7554/eLife.96458 (PMC13016608; doi:10.7554/eLife.96458)
Supplement: Supplementary file 1. — All strains were derived from the wild-type Bristol strain N2 and were cultivated at 20°C under standard conditions. Abbreviated names and full genotypes of the strains used in this study are listed here. [file elife-96458-supp1.docx]

| **Strain** | **Genotype** | **Reference in text** |
| --- | --- | --- |
| N2 | *C. elegans* var Bristol (N2)*.* | Wild type |
| AV477 | *dsb-2(me96)* II | *dsb-2* |
| AV280 | *unc-119(e2498) III; him-17(ok424) V;* *meIs5[unc-119(+) + him-17::GFP].* | *him-17::GFP* |
| CA1117 | *dsb-1(we11)* IV/*nT1[unc-?(n754) let-?]* (IV;V). | *dsb-1* |
| CB4088 | *him-5(e1490)* V | *him-5(e1490)* |
| CB6036 | *him-17(e2806)* V | *him-17* |
| KR5305 | *rec-1(h2875)* I | *rec-1* |
| NSV250 | *him-5(ddr43[him-5::3XHA])* | *him-5::3xHA* |
| NSV205 | *him-17(ddr37[him-17::3XHA])* V | *him-17::3xHA* |
| NSV485 | *eaIs4;him-17(ddr37[him-17::3XHA])* V | *him-17::3xHA;him-5::GFP::3xFLAG* |
| NSV508 | *gfp::dsb-1;him-5::3xHA* | *gfp::dsb-1;him-5::3xHA* |
| QP1102 | *rec-1(h2875)* I; *dsb-2(me96)* II | *dsb-2; rec-1* |
| QP1116 | *mre-11(iow1), him-5(e1490)* V/ *nT1 [qIs51]* ( (IV;V) | *him-5; mre-11* |
| QP1252 | *rec-1(h2875);cep-1(lg12501)* I | *rec-1; cep-1* |
| QP1317 | *rec-1 (h2875)* I;*him-17(e2806)/nT1g* V | *rec-1; him-17* |
| QP1366 | *parg-1(gk120)* IV;*him-5(ok1896)* V | *him-5; parg-1* |
| QP 1367 | *dsb-2(me96)* II;*parg-1(gk120)* IV | *dsb-2; parg-1* |
| QP1368 | *rec-1(h2875)* I*;parg-1(gk120)* IV | *rec-1; parg-1* |
| QP1370 | *cep-1(lg12501)* I*;parg-1(gk120)* IV | *parg-1; cep-1* |
| QP1373 | *dsb-2(me96)* II;*him-17(e2806)* V | *dsb-2; him-17* |
| QP1374 | *parg-1(gk120)* IV; *him-17(e2806)* V | *him-17; parg-1* |
| QP1550 | *cep-1(lg12501)* I;*dsb-2(me96)* II | *cep-1; dsb-2* |
| QP1572 | *parg-1(gk120)* IV;*mre-11(iow1)/nT1gU* [*unc-?(n754) let-?]* (IV; V) | *mre-11; parg-1* |
| QP1573 | *cep-1(lg12501)* I;*mre-11(iow1)/ nT1[qIs51]* (IV;V) | *cep-1; mre-11* |
| QP1623 | *rec-1(h2875)* I;*mre-11(iow1)/nT1gU* *[unc-?(n754) let-?]* (IV; V) | *mre-11; rec-1* |
| QP1624 | *dsb-2(me96)* II;*mre-11(iow1)/ unc-?(n754) let-?* (IV; V) | *dsb-2; mre-11* |
| QP1710 | *eaIs4[Phim-5::him-5::gfp::3xFLAG];him-17(e2707)* V | *eaIs4;him-17(e2707)* |
| QP1744 | *eaIs4[Phim-5::him-5::gfp::3xFLAG + unc-119(+)];him-17(ok424)* V | *eaIs4; him-17(ok424)* |
| QP1749 | *eaIs15[Ppie-1::him-5::gfp + unc-119(+)];him-17(ok424)* V | *eaIs15;him-17(ok424)* |
| QP1907 | *dsb-1(we11) IV/nT1 [qIs51]* (IV; V);*eaIs15[Ppie-1::him-5::gfp +unc-119(+)]* | *dsb-1/nT1; eaIs15* |
| QP1909 | *dsb-2(me96)* II;*eaIs15[Ppie-1::him-5::gfp +unc-119(+)];* | *dsb-2; eaIs15* |
| QP1961 | *eaIs4 (Phim-5::him-5::gfp::3xFLAG::him-5 3’ UTR + unc-119(+))* | *eaIs4* |
| PCM575 | *dsb-1(icm97[GFP::dsb-1])* IV | *GFP::dsb-1* |
| RB869 | *xnd-1(ok709)* III | *xnd-1* |
| RB1562 | *him-5(ok1896)* V | *him-5(ok1896)* |
| SSM2 | *mre-11(iow1)/nT1[qIs51]* (IV;V) | *mre-11* |
| VC130 | *parg-1(gk120)* IV | *parg-1* |
| VC255 | *+/nT1* IV*; him-17(ok424)/nT1* V | *him-17(ok424)* |
| XY1054 | *cep-1(lg12501)* | *cep-1* |
